# chemmodlab: a cheminformatics modeling laboratory R package for fitting and assessing machine learning models

**DOI:** 10.1186/s13321-018-0309-4

**Published:** 2018-11-28

**Authors:** Jeremy R. Ash, Jacqueline M. Hughes-Oliver

**Affiliations:** 1 Department of Statistics, Bioinformatics Research Center, North Carolina State University, 335 Ricks Hall, Campus Box 7566, Raleigh, NC 27695-7566 USA; 2 Department of Statistics, North Carolina State University, 2311 Stinson Drive, Campus Box 8203, Raleigh, NC 27695-8203 USA

**Keywords:** Machine learning, QSAR, R package, Initial enhancement, Enrichment factor, Accumulation curve, Hit enrichment curve, Repeated cross-validation

## Abstract

The goal of chemmodlab is to streamline the fitting and assessment pipeline for many machine learning models in R, making it easy for researchers to compare the utility of these models. While focused on implementing methods for model fitting and assessment that have been accepted by experts in the cheminformatics field, all of the methods in chemmodlab have broad utility for the machine learning community. chemmodlab contains several assessment utilities, including a plotting function that constructs accumulation curves and a function that computes many performance measures. The most novel feature of chemmodlab is the ease with which statistically significant performance differences for many machine learning models is presented by means of the multiple comparisons similarity plot. Differences are assessed using repeated *k*-fold cross validation, where blocking increases precision and multiplicity adjustments are applied. chemmodlab is freely available on CRAN at https://cran.r-project.org/web/packages/chemmodlab/index.html.

## Introduction

It is now commonplace for researchers across a variety of fields to fit machine learning models on complex data to make predictions. The complexity of these data (e.g., large number of features, non-linear relationships with the response) often means it is difficult to determine a priori what machine learning modeling routine and what descriptors (also known as features, predictors, or covariates) will result in the best performance. A common approach to this problem is to fit many descriptor set and modeling routine (D–M) combinations, and then compute measures of prediction performance for held out data to choose a D–M combination by assessing relative performance.

Sometimes in a particular domain, there are only a few modeling routines that are widely accepted, and researchers tend to use these methods exclusively. Unfortunately, this will not always work well for every data set and researchers might learn from other fields where different modeling methods tend to be more successful. There are a myriad of modeling methods implemented in R that may be worthwhile for researchers to try (see [1] and [2] for an overview of these methods). However, the lack of knowledge of the syntactic minutiae and statistical methodology that is required to fit and compare different modeling routines in R often prohibits users from attempting them.

chemmodlab [3] currently implements 13 different machine learning models. Fitting the entire suite of models requires little user intervention—all models are fit with a single command. Sensible defaults for tuning parameters are set automatically, but users may also tune models outside of chemmodlab (in caret [4], for example) and then manually set the tuning parameters.

While the R package caret has a similar goal, the fitting of many D–M combinations and the determination of statistically significant performance differences still requires some knowledge of R programming and statistical methods. chemmodlab further automates this process and provides several plotting utilities for easily assessing the results, lowering the barrier of entry for researchers unfamiliar with these methods.

The R package RRegrs [5] automates the process of fitting and assessing many cheminformatics regression models. Their pipeline also performs automated descriptor preprocessing, repeated cross validation, assessment of applicability domain, and provides tools for visualizing model performance measures. The R package camb [6] provides an alternative cheminformatics regression modeling pipeline. Some of the novel contributions of this package are tools for descriptor calculation, exploratory data analysis, and ensemble modeling. chemmodlab complements these packages by providing novel methods for model fitting and assessment in both regression and classification frameworks. We have placed particular emphasis on implementing classification model assessment methods that directly address the aspects of prediction performance that are of interest in cheminformatics (e.g., initial enhancement, accumulation curves). Again, the most novel aspect of chemmodlab is the means by which statistically significant differences in these performance measures are computed and visualized for many D–M combinations.

chemmodlab may also be useful in non-chemical applications, and we regard this to be a strength rather than a weakness. This package, however, was directly motivated by a need to assess machine learning models that relate chemical structure to observed endpoints using criteria relevant for, and catered to, cheminformatic applications. It is availability of these assessment criteria that uniquely defines this package as cheminformatic.

One motivation for this package was the observation that once performance measures are computed for several different D–M combinations, researchers often do not consider the randomness and uncertainty involved in obtaining the observed performance measures. If one model has prediction performance that is marginally better than another, it is tempting to claim improvement. However, *very slight* changes in the original data set or in how assessment was conducted could have led to a reversal of observed performance. By accounting for the inherent uncertainty in data collection and model assessment, a stronger and more defensible claim can be made about differences in prediction performance. For example, a carefully constructed confidence interval that does not contain zero for the difference in performance measures between two D–M combinations would reliably identify significant differences between the two D–M combinations, even after accounting for uncertainty.

**Fig. 1:**
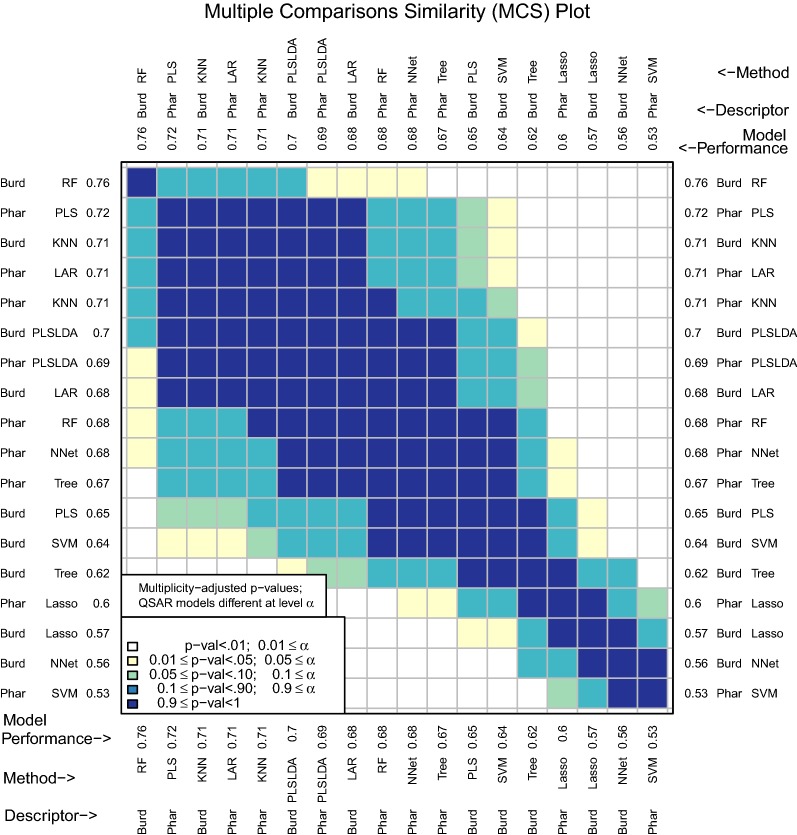
MCS plot using area under the receiver operating characteristic curve as the performance measure

Figure 1 shows an example of this. Many classification models have been fit to two different descriptor sets to predict a binary response variable. There are a total of 18 D–M combinations to be compared. The D–M combinations were assessed using repeated tenfold cross validation and the area under the receiver operating characteristic curve (AUC) performance measure. A multiple comparisons similarity (MCS) plot visualizes the differences in model performance (Fig. 1). The descriptor sets will be discussed in detail later on, but for now, it is sufficient to say that the Pharmacophore descriptors are far more interpretable than the Burden Number descriptors. The Burden Numbers-Random Forest (RF) combination is the best performing D–M combination (AUC: .76). However, the Pharmacophore-Least Angle Regression (LAR) combination (AUC: .71) involves a highly interpretable linear model with a subset of the Pharmacophore descriptors selected. This .05 difference is small and without additional investigations it is unclear whether it is statistically significant.

By performing multiple cross validation splits and using these splits as a blocking factor to improve precision, chemmodlab is able to test for statistical significance of performance measure differences and visualize these results in a manner that can be easily interpreted by the user. The question this addresses is: if the experiment were repeated with changes to the training and/or test set, would the best performing model still be the best? Again referring to Fig. 1, the MCS plot indicates that a significance level of 0.01 leads to the conclusion that the two aforementioned D–M combinations (Burden Numbers-RF, and Pharmacophores-LAR) are equivalent, and hence the more interpretable model may be selected for further investigations. This inference has been multiplicity-adjusted for the ${18 \atopwithdelims ()2} = 153$ pairwise comparisons that were made.

chemmodlab is a re-creation and extension of the former ChemModLab webserver [7]. Some notable extensions to the previous software are:

- chemmodlab has been redesigned so that it is usable in the R environment. Models are fit with a simple command, producing an object that can be easily passed to plotting and performance assessment functions;
- much more control over the model fitting and assessment functions. There are now many arguments for customizing the procedures and the output they provide, including the appearance of the plots generated. As an example, chemmodlab now has support for user supplied tuning parameters so that models can be better fit to the data at hand;
- many new model performance measures for classification and regression have been implemented;
- and functions for computing molecular descriptors and applicability domain have been added;

chemmodlab is organized into two successive components: (1) model fitting, which is primarily conducted via function *ModelTrain*, and (2) model assessment, which is conducted via function *CombineSplits*.

## Results and discussion

### Preparing the data

We will use a cheminformatics data set to illustrate a typical analysis pipeline in chemmodlab. Our goal is to build machine learning models that relate a chemical’s structure to an observed endpoint. These models are generally referred to as quantitative structure–activity relationship (QSAR) models. See [8] for an excellent review of the ways in which these models have played a crucial role in the drug discovery process. Often the “endpoint” (or response variable) is a measure of a compound’s biological activity, which may be either binary, indicating active/inactive, or a continuous measure, e.g., representing binding affinity for a target protein. Chemical descriptors represent various levels of organization of a chemical’s structure. See [8] for an overview of commonly used chemical descriptors.

**Fig. 2:**
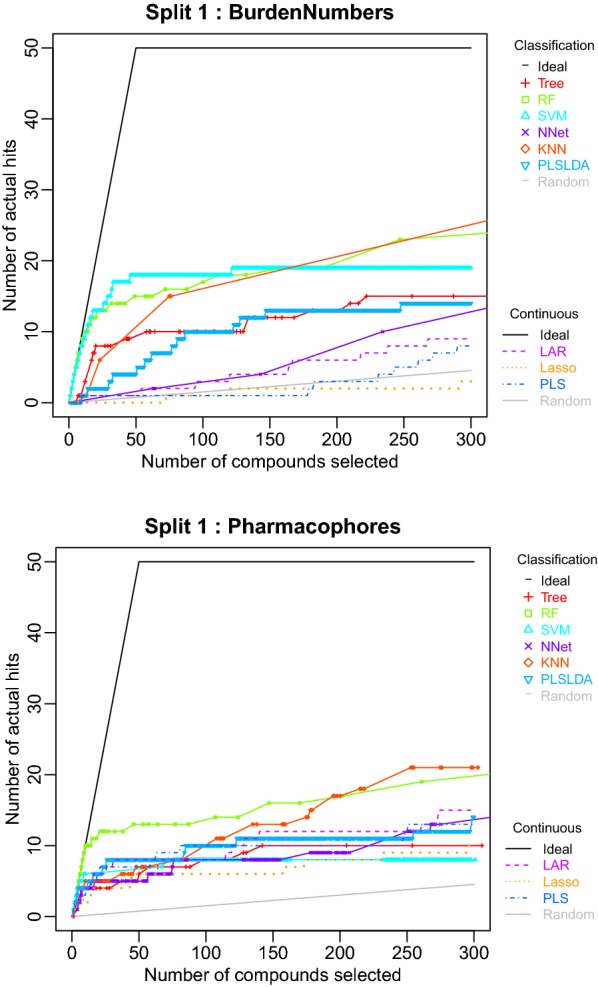
Comparison of the first cross validation split accumulation curves for models fit to the Burden Numbers descriptor set (top) and the Pharmacophore descriptor set (bottom)

The data we will analyze is from a cytotoxicity assay conducted by the Scripps Research Institute Molecular Screening Center. There are 3,311 compounds, 50 of which are active. Visit the website [9] for more details.

For completeness, the preprocessing of the data set analyzed will be shown. First, the response variable and molecule IDs are read in from file.

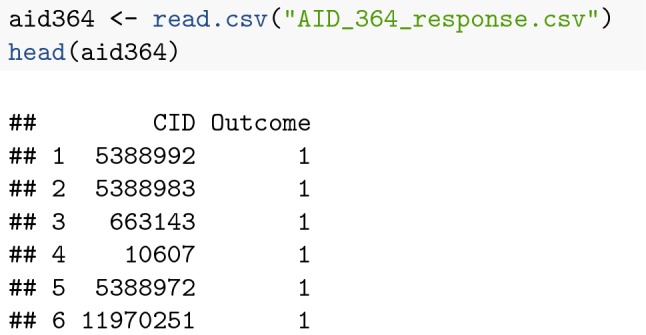



Next, two descriptor sets are added to the data frame. Both of these sets were computed using the software PowerMV—see [10] for more information. The first set of 24 continuous descriptors are a modification of the Burden number descriptors [11].

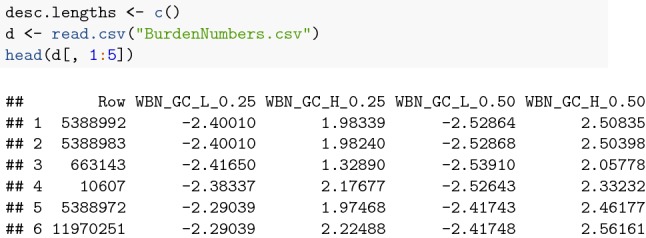

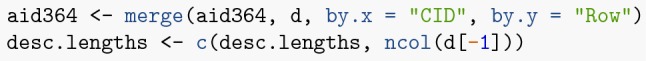





The number of descriptors in each descriptor set are also stored, as this information will be used to parse the data frame later on. The second descriptor set contains 147 binary descriptors, indicating the presence/absence of “pharmacophore” features, described in more detail in [10].

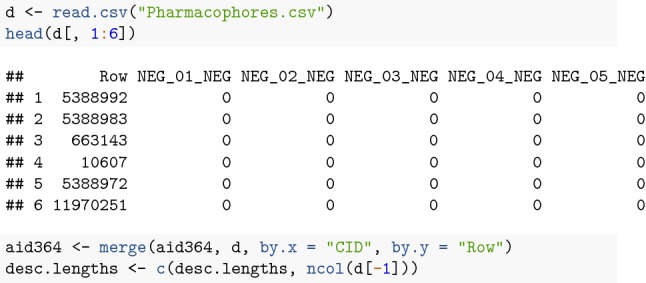



A subset of this data set, containing all 50 active compounds and an additional 450 compounds, is included in chemmodlab.

### Model fitting: the *ModelTrain* function

For the model fitting component of chemmodlab, the primary function is *ModelTrain*, which fits a series of machine learning models to a data set.

Function *ModelTrain* takes as input a data frame with an (optional) ID column, a response column, and descriptor columns. We have processed the *aid364* data set so that it follows this format. The specification of an ID column allows users to easily match response predictions to their observation IDs in the chemmodlab output.

chemmodlab can currently handle responses that are continuous or binary (represented as a numeric vector with 0 or 1 values). Assessment assumes preference for large response values. Users can specify which columns in the data frame they would like to consider as distinct descriptor sets. At the moment, the response and descriptors may only be binary or continuous, though we are currently working on support for categorical variables of more than two levels.

For our example, we previously stored the number of descriptors in each descriptor set in an integer vector named *desc.lengths*, with the ordering of the integers matching the order of the descriptor sets in *aid364*:

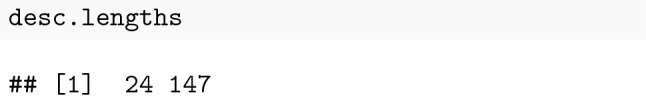



Users can also name the descriptor sets by providing a character vector to the *des.names* argument. If this character vector is specified, all of *ModelTrain* output and downstream chemmodlab functions will name the descriptor sets accordingly:

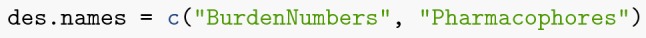



The specification of distinct descriptor sets in a data frame is illustrated in the following call to *ModelTrain*:

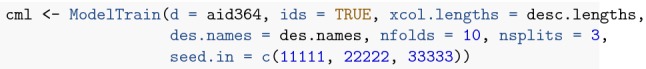



The *nsplits* argument sets the number of splits to use for repeated validation and *nfolds* sets the number of folds to use for each cross validation split. The default values have been used. *seed.in* sets the seeds used to randomly assign folds to observations for each repeated cross-validation split. If NA, the first seed will be 11111, the second will be 22222, and so on.

If the descriptor set columns are not identified by the user, *ModelTrain* assumes there is one descriptor set, namely all columns in d except the response column and optional ID column. Alternatively, the argument *xcols* may be used to explicitly specify the columns corresponding to each descriptor set. Also, if it is more convenient, descriptor sets can be provided as a list of matrices with the argument *x* and the response as a numeric vector with the argument *y*.

### Descriptor set creation

In the event that a user has not precalculated descriptors, chemmodlab can compute descriptor sets for use in model building, based on chemical structures provided by the user. *ModelTrain* accepts molecule objects that are created by the package rcdk [12] that supports most of the widely used chemical file formats (SMILES, SDF, InChI, Mol2, CML, etc.). When molecules are provided to *ModelTrain*, the names of predefined descriptor sets and/or fingerprints must also be provided. *ModelTrain* then calls either rcdk or the package fingerprint [13] to compute the requested descriptors sets for the molecules provided. rcdk computes these descriptors by interfacing with the chemistry development kit (CDK) [14, 15].

We use the *bpdata* data set [16, 17] provided by the rcdk package to illustrate. The first column of *bpdata* contains 277 chemical structures in SMILES format, with a majority of the molecules being alkanes and substituted alkanes. The second column of *bpdata* contains boiling points, the endpoint we would like to predict.

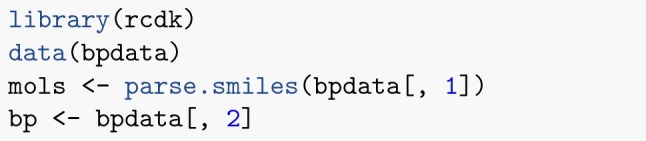



*ModelTrain* may now be given the molecule object, *mols*, along with a list of molecular descriptor categories to be generated by CDK:

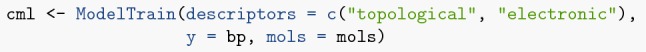



Users may also provide any of the 11 fingerprint types computed by the fingerprint package [13].

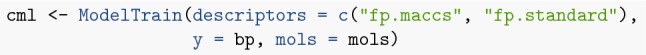



### Model fitting: chemmodlab models

Currently, 13 different machine learning models are implemented in chemmodlab. The details of each modeling method, including descriptions of the default parameters, are provided at https://jrash.github.io/chemmodlab/. Briefly, the current models are: elastic net (ENet), k-nearest neighbors (KNN), lasso (Lasso), least angle regression (LAR), neural networks (NNet), partial least squares linear discriminant analysis (PLSLDA), partial least squares (PLS), principal components regression (PCR), ridge regression (Ridge), random forest (RF), two implementations of recursive partitioning (Tree, RPart), and support vector machines (SVM).

These models have been carefully chosen to provide good coverage of the spectrum of model flexibility and interpretability available for models implemented in R. Typically, models that are more flexible (e.g., NNet) are capable of fitting more complex relationships between predictors and response, but suffer in terms of model interpretability relative to other less flexible models (e.g., Lasso). By testing for significant differences between model performance measures, we hope to find interpretable models whose performance is not significantly different from (i.e., plausibly equivalent to) the best model, suggesting a model that provides an understanding of the relationship between the predictors and response that exceeds mere prediction.

Some modeling strategies may not be suitable for both binary and continuous responses. Six of the models have implementations in R that directly support both binary and continuous responses (Tree, RPart, RF, KNN, NNet, and SVM). However, six methods (Lasso, LAR, Ridge, ENet, PCR, and PLS) assume that responses have equal variances and normal distributions. This assumption is often reasonable for continuous responses, but may be suspect if the response is binary. For these latter six methods, binary responses are treated as continuous, resulting in continuous response predictions that are not restricted to range between 0 and 1. A threshold can then be applied to obtain a binary predicted response. The model assessment functions discussed later allow users to select this threshold. Finally, PLSLDA cannot be applied to a continuous response, but if the user wishes to analyze this type of data, a threshold value may be used to convert a continuous response to a binary one.

In cheminformatics applications, descriptors often show strong multicollinearity. Since this is often problematic for machine learning models, we have specifically included several models in chemmodlab that are known to be resilient to multicollinearity (e.g., PCR and PLS). However, with the exception of PCR, which utilizes uncorrelated linear combinations of the original descriptors and is a highly interpretable model, models for which prediction is not considerably affected by multicollinearity do suffer in terms of model interpretability. For example, when one variable of a set of highly positively correlated variables is selected for inclusion in the Lasso linear model, the selection of the variable is essentially arbitrary. Fitting the same Lasso model to a slightly different data set would likely result in the selection of a different variable from the same set.

chemmodlab has been designed in a way that it is easily extensible to new machine learning modeling methods, and new modeling methods will be added as the authors identify those that have broad utility to our users. Support for other models can be requested here: https://github.com/jrash/chemmodlab/issues.

chemmodlab automatically performs data preprocessing before fitting the models that require it (e.g., centering and scaling variables before PCR), so the user need not worry about preprocessing of descriptors prior to model fitting.

### Model fitting: specifying model parameters with *user.params*

Sensible default values are selected for each tunable model parameter, however users may set any parameter manually using the *user.params* argument.

*MakeModelDefaults* is a convenience function that makes a list containing the default parameters for all models implemented in *ModelTrain*. Users may set any parameter manually by generating a list with this function and modifying the parameters assigned to each modeling method:

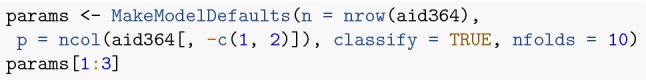

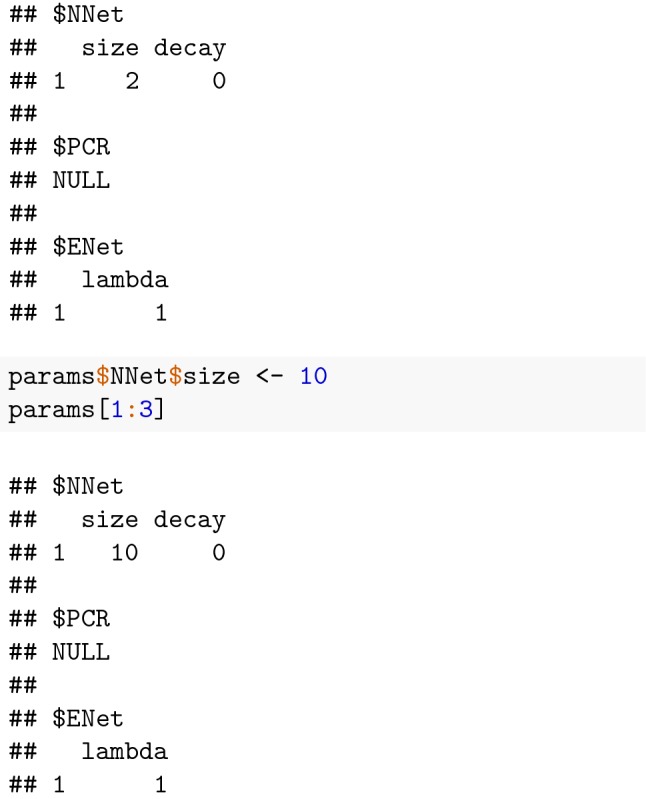





This list can then be provided to the *user.params* argument to assign the tuning parameter values used by *ModelTrain*:

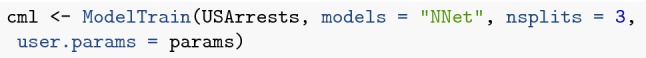



### Model assessment: repeated *k*-fold cross-validation

For each descriptor set, *ModelTrain* performs repeated *k*-fold cross validation for the selected set of machine learning models.

For each cross-validation split, observations are randomly assigned to one of *k* folds, splitting the data set into *k* blocks that are approximately equal in size. The number of cross validation folds (*k*) is set with the *nfolds* argument. Users may also use the *seed.in* argument to set the seed for each split, so that the *ModelTrain* results are reproducible. Each block is iteratively held out as a test set, while the remaining $k-1$ blocks are used to train each D–M combination. Predictions for the held out test set are then made with the resulting models.

Many resampling methods for assessing model performance involve partitioning a data set into a training set and test set. With these methods, predictions are made on observations that are not involved in training. This results in model performance measures that are less likely to reward over-fitting.

Since performance measures can be highly variable [18] depending on which observations are held out and which are involved in training, the repetition of this procedure during *k*-fold cross validation and the averaging of the performance measures often result in a more accurate estimation of model performance than a one-time split.

Finding the right number of cross-validation folds for the estimation of a performance measure involves consideration of the bias-variance trade off. The mean squared error of an estimator, a measure of how accurately an estimator estimates the true value of a parameter, can be partitioned into two components, bias and variance:

$$\begin{aligned} E[(\hat{\theta } - \theta )^2] = (E[\hat{\theta }] - \theta )^2 + Var[\hat{\theta }], \end{aligned}$$

where $\hat{\theta }$ is the estimator of the true performance measure, $\theta$, for the population of test sets similar to the data set under consideration. The first component is squared bias and the second is variance. An increase in either the bias or variance will decrease the quality of an estimator. When a resampling method substantially over- or under-estimates a performance measure on average, it is said to have high bias. Bias is often related to size of the data set that is held out as a test set [18]. The smaller the number of folds in *k*-fold cross validation, the more observations are held out in each fold, and the less observations that are used to train a model. Fewer observations in a training set means that a model is likely to perform worse, and model predictions tend to miss the target. Thus, performing *k*-fold cross validation with two folds, where there is 50% of the data in each fold, would likely result in high bias.

In contrast, a performance measure estimator suffers from high *variance* when its estimate varies considerably when there are slight changes made to the training and/or test set. Leave-One-Out-Cross-Validation (LOOCV) refers to *k*-fold cross validation with *k* equal to $n$, the number of observations. LOOCV often suffers from high variance [18]. This is due to the fact that the training set changes very little with each iteration of LOOCV. Thus, performance measure estimates tend be highly positively correlated. The mean of a highly correlated variable has higher variance than an uncorrelated one. Decreasing the number of folds tends to decrease the correlation of performance measure estimates and lower the variance. Therefore, the ideal number of folds to use for cross validation is often somewhere between 2 and $n$. The number of folds often used for *k*-fold cross validation are 5 and 10, as these values frequently provide a good balance between bias and variance.

Several studies [19–21] have shown that repeated cross validation can reduce the variance for a *k*-fold cross validation procedure with low bias, achieving a more accurate estimation of model performance. When *k*-fold cross validation is repeated in chemmodlab, multiple iterations of random fold assignment, or “splits”, are performed. Because the observed performance measures may vary greatly depending on the definition of folds during *k*-fold cross validation, all models are built using the same fold assignment, thus using fold definition as a “blocking factor” in the assessment investigation. This process is repeated for multiple *k*-fold cross validation runs. The user may choose the number of these splits with the *nsplit* argument.

There are a myriad of potential model violations that may lead to over- or under-estimation of model performance measures. These include but are not limited to: a misspecification of the structural form of the model (e.g., more descriptors, or a function of the descriptors should be used), correlation between responses, measurement error in predictors, non-constant variance in the response, collinearity between descriptors, and non-normality of the response. Also, the types of model violations that occur in the training and test sets generated by *k*-fold cross validation may vary depending on how the data is split, resulting in over- or under-estimation of a performance measure depending on how the data was split. Since it is implausible to account for all model violations, our approach is to treat the effect of model violation as a nuisance variable. By doing several random splits and averaging the *k*-fold cross validated performance measures, we “average out” the model violation effect on the performance measure estimate.

Some authors [22–24] argue that external validation is necessary to determine the predictive power of a QSAR model, and that cross validation is not sufficient to assess prediction performance. First, we would like to be careful not to conflate cross validation prediction performance and training set prediction performance. Training set prediction performance assesses predictions on data used in model training (also referred to as model building or fitting), and this will often be overly optimistic. Cross validation provides a less biased estimate of prediction performance on new data [25, 26]. This is because in each iteration, cross validation holds out a fold of data, trains the model on the remaining data, and makes predictions on the held out fold; in this sense, cross validation does use external data for assessment of predictive performance. In order for cross validation to provide an accurate estimate, it is essential that every step of a multistep modeling procedure is performed within the cross validation procedure. This means that the “held out” folds must be removed before any variable selection or data filtering is performed. See [1] for a careful discussion.

There are numerous reasons why *k*-fold cross validated model performance measures may not agree with those obtained by external validation, as was observed in [22]. As previously discussed, estimates of model performance measures from a single split in the data often suffer from high variance, resulting in a poor estimate of the true prediction performance of a model on new data, particularly when the data sets are small [27]. This was the case in [22], but chemmodlab can ameliorate this because multiple splits are run by default.

Another common cause of “over-confident” *k*-fold cross validation is that variable selection is not performed within the cross validation process. This will lead to overfitting and over-confident performance measures [27, 28]. This may, in part, explain the over-confident $q^2$ values observed in [22]. The task of variable selection (i..e., identification of the most important descriptors) often follows the task of model selection. Strictly speaking, chemmodlab is focused on the task of model selection, but its strength in identifying statistically equivalent models can also provide guidance in variable selection. For example, chemmodlab could conclude statistical equivalence between D–M combination *A* and D–M combination *B*, where *A* has far fewer and more interpretable descriptors than *B*. The practitioner would likely choose to use D–M combination *A*, thus simultaneously conducting model selection and variable selection. Additionally, some of the models in chemmodlab automatically perform variable selection (e.g., Lasso, ENet). Most importantly, chemmodlab carefully imbeds the entire selection process within repeated cross validation, so variable selection happening outside of the cross validation process is not an issue.

The assumptions of *k*-fold cross validation may also be violated. When building prediction models, all methods assume that the training data is representative of real-world data; if this assumption fails, all hope is lost. Another assumption of *k*-fold cross validation is that excluded compounds are independent of compounds that remain in the set. Although this is often a reasonable assumption, replication and pseudo-replication could invalidate this assumption. QSAR models are often developed for compounds in an analog series, where blocks of compounds have highly similar descriptor values. Xu et al. [29] demonstrate dramatic effects of properly addressing this assumption by shifting from leave-one-out cross validation (which suggested optimistic model performance) to leave-one-solute-out cross validation (which removed dependencies and consequently was more similar to results from external validation). To incorporate the Xu et al. [29] adjustments, the user would need to provide more input to chemmodlab for describing the experimental setup, and we are considering a future package update to handle this. In the meantime, our multiple runs of *k*-fold cross validation may sufficiently break the dependency patterns, thus indirectly correcting for a possibly invalid assumption.

The ideal scenario for assessing predictive performance of a model is to have a very large training set that may be used for internal validation, plus several large sets that may be used for external validation. Because resources often make this impossible, the use of an effective and efficient internal validation strategy is critical [1]. *k*-fold cross validation has been shown to be both an effective and efficient method for assessing predictive performance of models [25, 26, 30–34].

For external validation, it would be ideal to have a large *independently* collected data set to assess how well a predictive model generalizes to data collected under different conditions (e.g., collected from different labs or under different experimental conditions) [35]. However, a data set like this is often not available. Often, what researchers call external validation is equivalent to a single random split of a small data set. In this scenario, repeated *k*-fold cross validation will likely provide users with a more accurate estimate of model performance. As explained previously, *k*-fold cross validation averages performance measures from several splits in the data, resulting in a reduction of variance of the model performance estimate [34].

We will see later how, in addition to increased accuracy in estimation of performance measures, repeated cross validation allows one to measure the standard error of model performance measures, quantifying how sensitive performance measures are to fold assignments.

### Model assessment: accumulation curves

*plot.chemmodlab* takes a chemmodlab object output by the *ModelTrain* function and creates a series of accumulation curve plots for assessing model performance.

The accumulation curve (or hit enrichment curve) for binary responses shows the number of positives versus the number of “tests” performed, where testing order is determined by the *k*-fold cross validated predicted probability of a response being positive. The *max.select* argument sets the maximum number of tests to plot for the accumulation curves. By default, $\left\lfloor {{\min (300, \frac{n}{4})}}\right\rfloor$ is used, where $n$ is the number of observations. This prioritizes finding actives in a relatively small number of tests.

Two series of plots are constructed. In the “descriptor” series, there is one plot per cross validation split and descriptor set combination; the accumulation curves for each modeling method are plotted together so that they can be compared. In the “methods” plot series, there is one plot per cross validation split and modeling method combination; the accumulation curves for each descriptor set are plotted together so that they can be compared.

By default, a large number of accumulation curve plots are constructed. The *splits* argument may be used to only plot a subset of splits. The *meths* argument may be used to only plot a subset of methods in the “methods” series. The *series* argument specifies whether the “methods” series of plots, the “descriptors” series of plots, or both are generated.

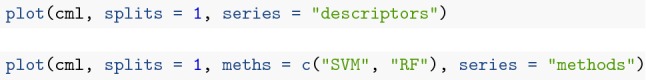



An “ideal” curve is plotted on these graphs to demonstrate the accumulation curve of a model that correctly identifies the *p* positives in the first *p* tests. Thus, at *m* tests, models with accumulation curves that are nearest to the ideal curve are preferable. Also, if an accumulation curve has a slope that is parallel to the ideal curve for an interval of tests, the model has ideal performance for that interval. A “random” curve shows the accumulation curve if the testing order were decided at random. At *m* tests, models with accumulation curves that are below the random curve have worse performance than random chance. Models that were fit as classification models (see “Model fitting: chemmodlab models”) are represented as solid lines with different colors and shapes specifying the modeling method. Models that were fit as continuous models and then thresholded are differentiated by line type and color.

In Fig. 2, we have plotted the accumulation curves for the first cross validation split, generating two plots in the “descriptors” series, one for each descriptor set. Comparing the accumulation curves for models utilizing Burden Number descriptors, SVM and RF have much better hit rates than the other models for the initial 100 compounds prioritized for testing. However, if more than 100 tests were to be performed, the KNN method would have the best performance. Intersections between model accumulation curves indicate the number of tests at which one model’s performance overtakes another’s. Considering the Pharmacophore fingerprints, the RF method has ideal performance initially, but is eventually superceded by the KNN method, as was the case for the Burden Number descriptors.

**Fig. 3:**
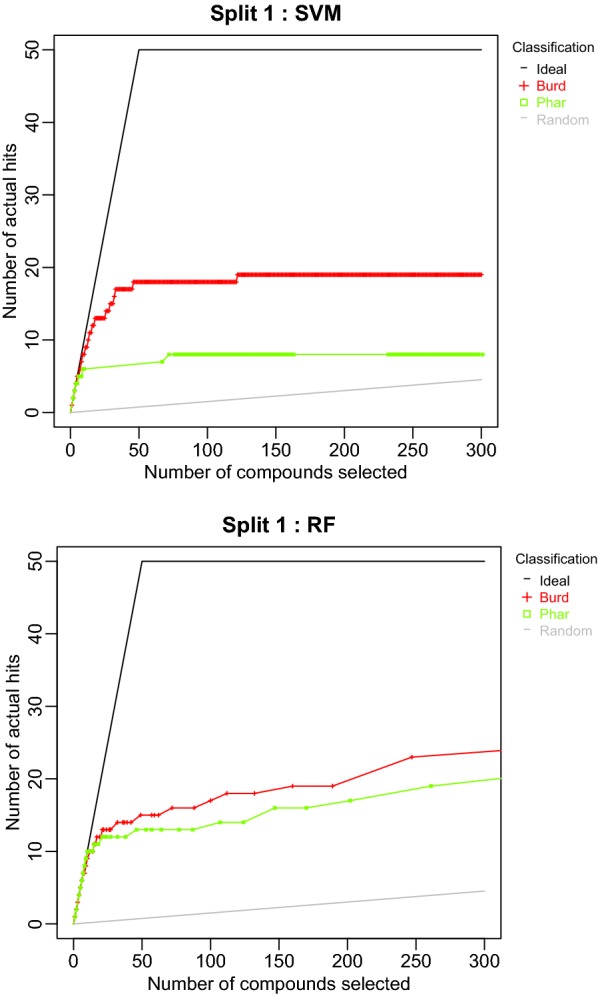
Comparison of the first cross validation split descriptor set accumulation curves for the SVM method (top) and the RF method (bottom)

The next series of plots (Fig. 3) is the “methods” series. When the Split 1 descriptor set performances are compared for the SVM method, the Pharmacophore fingerprints have considerably worse performance than the Burden Numbers. This is sensible as SVM performance often suffers in high dimensional spaces. Comparing descriptor set performances for the RF method, the Burden Number descriptors have slightly improved performance over the Pharmacophore fingerprints for all but the initial few tests. However, at any number of tests at which Burden Numbers outperform Pharmacophores, Burden Numbers only provide a few more hits. Therefore, it is plausible that if this experiment were performed again with a different, but similar data set, Pharmacophores would perform equally as well as Burden Numbers. One may be concerned about the statistical significance of this improved performance. This observation has motivated the construction of the *CombineSplits* function, which rigorously tests for statistically significant differences.

The accumulation curve has also been extended to continuous responses. In QSAR models, a continuous response is often a measure of binding affinity (e.g., pKi) where a large positive value is preferable. Therefore, in these accumulation curves, testing order is determined by ordering the predicted response in decreasing order. The response is then accumulated so that $\sum _{i=1}^{m} y_i$ is the sum of the $y$ over the first *m* tests. The binary response accumulation curve is a special case of this.

### Model assessment: multiple comparisons similarity plot

*CombineSplits* evaluates a specified performance measure across all splits. This function assesses how sensitive performance measures are to fold assignments, or small changes to the training and test sets. Intuitively, this assesses how much a performance measure may vary if predictions were made on a test set that is similar to the data set analyzed. Multiplicity-adjusted statistical tests are used to determine the best performing D–M combination.

As input, *CombineSplits* takes a chemmodlab object produced by the *ModelTrain* function. *CombineSplits* can use many different performance measures to evaluate binary classification model performance (namely: error rate, sensitivity, specificity, area under the receiver operating characteristic curve, positive predictive value also known as precision, F1 measure, and initial enhancement). By default, *CombineSplits* uses initial enhancement proposed by [36] to assess model performance. Initial enhancement is also known as the hit enrichment factor in the cheminformatics literature. Initial enhancement at *m* tests is the hit rate—the fraction of accumulated positives at *m* tests—divided by the proportion of positives in the entire data set. This is a measure of a model’s hit rate fold improvement over random chance. A desirable model will have an initial enhancement much larger than one. A typical number of tests for initial enhancement is $m=300$.

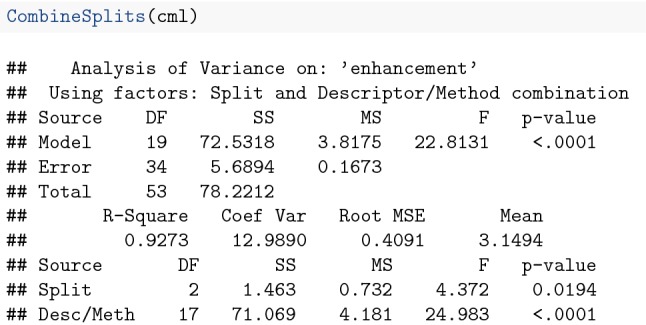



An advantage of performing repeated *k*-fold cross validation with *ModelTrain* is that the output can be viewed as a designed experiment with two factors: D–M combination and split (fold assignment). Therefore, *CombineSplits* performs an analysis of variance (ANOVA) to identify significant differences between the mean performance measures according to factor levels. The linear model corresponding to this ANOVA is:

$$\begin{aligned} Y_{ij} = \mu + \alpha _i + \beta _j + \epsilon _{ij}, \end{aligned}$$

where $\alpha _i$ corresponds to ith level of the split factor and $\beta _j$ to the jth level of the D–M combination factor. From the ANOVA table in this example, the split main effect is marginally significant (*p*-value of 0.0194), indicating that there is a significant difference between mean initial enhancement across splits, averaging over D–M combinations. In other words, some splits result in significantly larger initial enhancement values than other splits. This endorses our decision to treat splits as a blocking factor. The D–M combination main effect is highly significant (*p*-value $\le$ 0.0001). The “Error MS” estimates the variance in the performance measures within the groups corresponding to each combination of factor levels. The “Model MS” estimates the variance between groups.

**Fig. 4:**
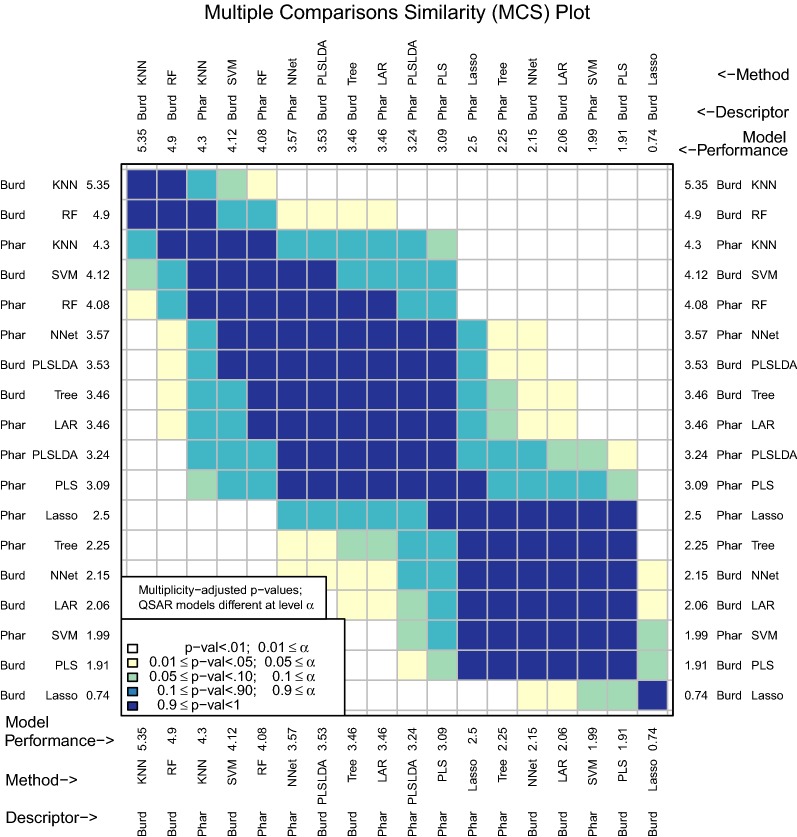
MCS plot using initial enhancement at 300 tests as the performance measure

The multiple comparisons similarity plot in Fig. 4 shows the results for tests of significance among all pairwise differences of mean model performance measures. Along both the x- and y-axes, D–M combinations are ordered from best to worst performance measure. Because there can potentially be a large number of methods (18 in the example leading to ${18 \atopwithdelims ()2} = 153$ pairwise comparisons), an adjustment for multiple testing is necessary. We use the Tukey–Kramer multiple comparisons procedure (see [37] and [38]).

In Fig. 4, Burden Numbers-KNN, Burden Numbers-RF, Pharmacophore-KNN, and Burden Numbers-SVM are the top performing models. We conclude that for the population of compounds that are similar to the data set analyzed, the initial enhancement of these four models is plausibly the same. The intuition is that if predictions were made on a new test set that is similar to the data set under consideration, these four models would plausibly have very similar prediction performance.

There are different characteristics of the top performing models that may lead to a researcher choosing one over the other. While KNN is not the most interpretable model, there are fast heuristic KNN regression methods implemented in R such as FNN [39] that make predicting on large data sets quite manageable. RF has a time complexity comparable to the fast heuristic KNN, yet also results in a more interpretable model. Measures of variable importance can be computed for RF, which allow users to identify the subset of variables that are most important for prediction. The Burden Numbers-SVM model is the least interpretable of the set and has time complexity that scales the worse with $n$ (between $O(n^2)$ and $O(n^3)$ time complexity). Though the Burden Numbers-KNN model is the best performing model according to mean initial enhancement, this model performance measure is only .45 larger than the Burden Numbers-RF model and the difference is not statistically significant. Since the Burden Numbers-RF model is more interpretable and works just as well with large data, this modeling method may be preferable.

The Pharmacophore-RF model is only significantly different from the best performing model at a level between .01 and .05, but may be preferrable, as both the descriptor set and the model are highly interpretable.

**Fig. 5:**
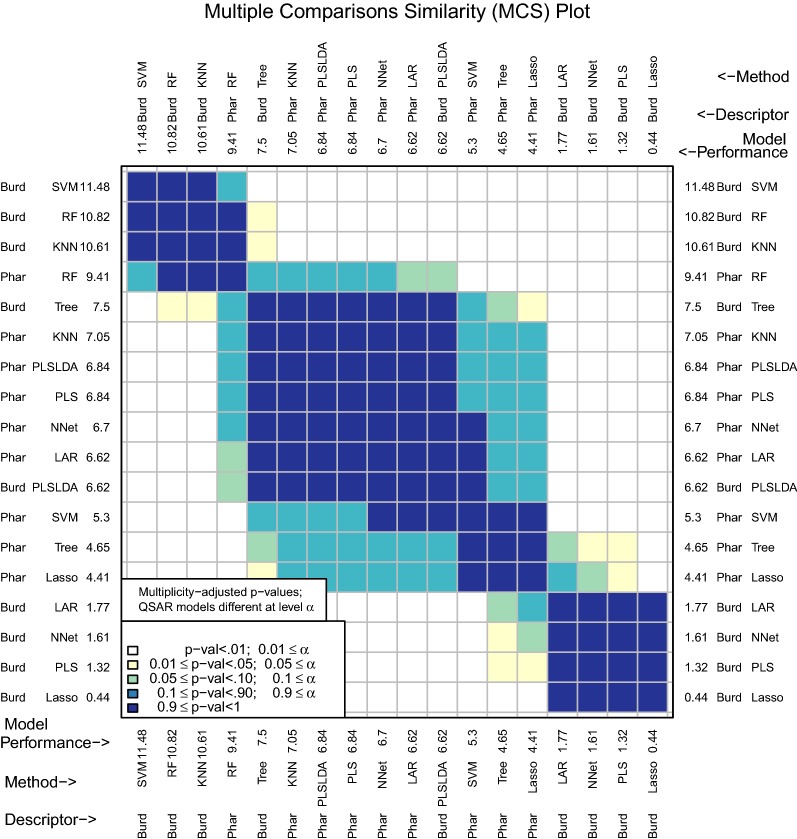
MCS plot using initial enhancement at 100 tests as the performance measure

For many applications, users may know the number of tests they would like to perform. This is often the case in drug discovery when chemists have a set number of compounds they would like to assay and the goal is to enrich this set of compounds with as many actives as possible. The number of tests used for initial enhancement may be modified with the *m* argument (Fig. 5):

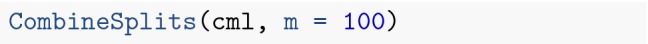



Figure 5 underscores the practical importance of using an appropriate number of tests for initial enhancement. If a significance level of .05 were to be used, the Pharmacophore-KNN model is no longer among the best performing models at $m=100$, while the highly interpretable model, Pharmacophore-RF, is now among the best. This model is the clear choice if an interpretable model is desired.

These results support the observations regarding descriptor set accumulations curves for the RF model in Fig. 3. While there appeared to be a slight improvement using Burden Number descriptors in lieu of Pharmacophore fingerprints, this improvement may not be considered statistically significant.

**Fig. 6:**
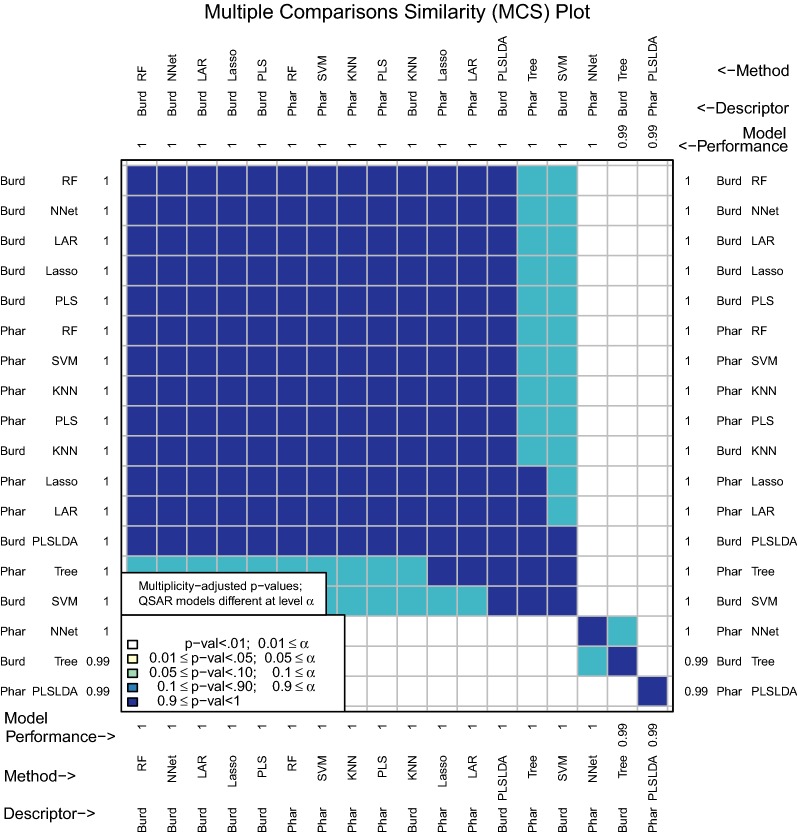
MCS plot using specificity for all compounds as the performance measure

In this particular example, specificity does not do a good job at distinguishing the best performing models, as the set of plausibly best performing models is quite large (Fig. 6). This is due to the fact that there are many models that have an average specificity that is similar to the best performing model:

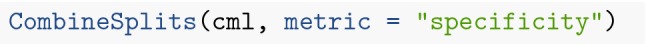



**Fig. 7:**
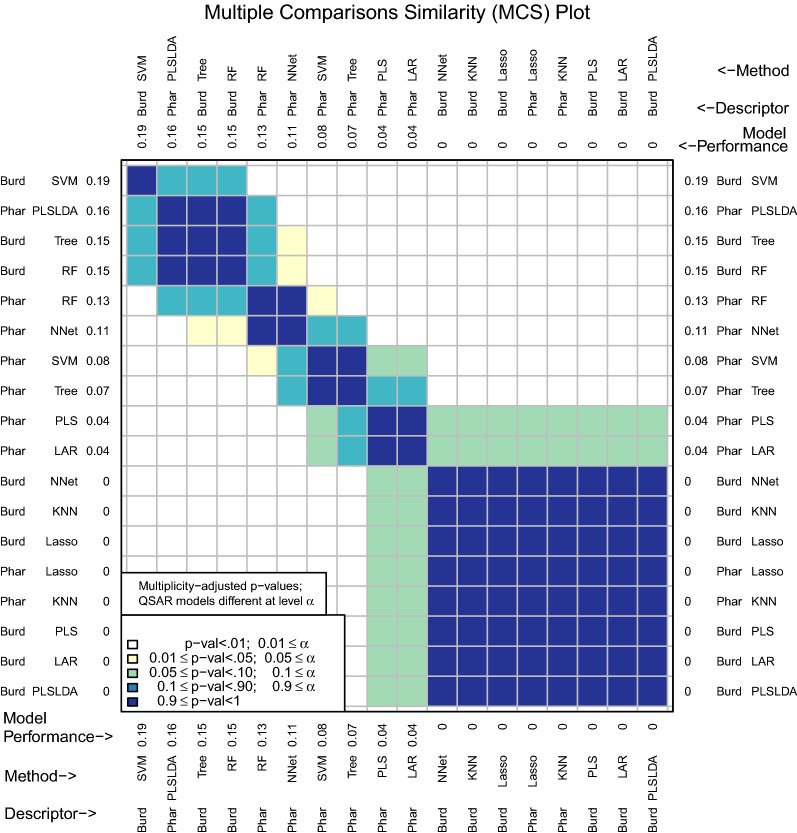
MCS plot using sensitivity for all compounds as the performance measure

Sensitivity, however, distinguishes the best performing model much better (Fig. 7):

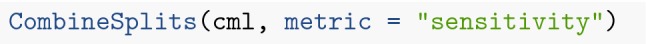



**Fig. 8:**
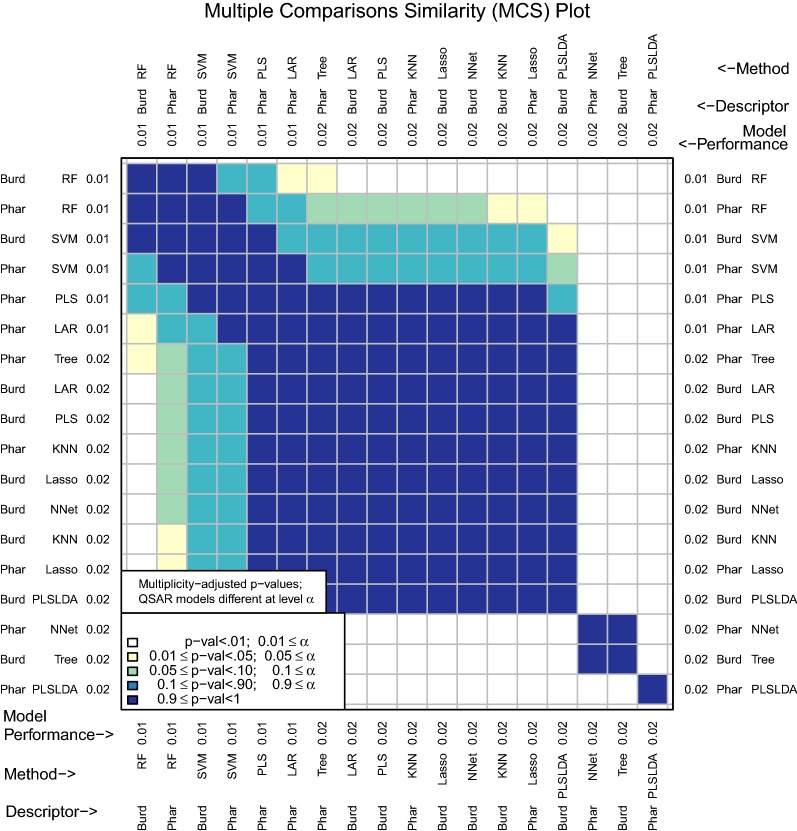
MCS plot using error rate for all compounds as the performance measure

For binary responses, model performance may also be assessed with misclassification rate (Fig. 8). However, this measure may be inappropriate for drug discovery because it equally penalizes false positives and false negatives. As we will explain in detail later on, researchers in cheminformatics tend to be less concerned with false negatives and instead prioritize finding actives early. Therefore, a measure like initial enhancement may be more appropriate.

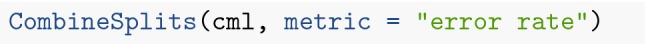



This example illustrates how using misclassification rate can be misleading if this is the only model performance measure. Misclassification rate suggests several models (such as Pharmacophore-PLS and Pharmacophore-LAR) are among the best performing models, but these models actually have very low sensitivity (both .04). These models identify only a few of the 50 true positives in the data set. If the goal of a medicinal chemist is to identify all of the active compounds in their data set, these models may actually have poor performance.

It is also possible that models with high sensitivity incorrectly identify many compounds as active. The positive predictive value (PPV), also known as precision, measures the percentage of compounds that were correctly predicted to be active. PPV is an important means of evaluating search engines. A search engine often finds a multitude of potentially relevant documents, but a user is only capable of looking at the first few results. Search engines need to have very high PPV, even at the expense of sensitivity and specificity [40]. The goal is not to correctly identify all the relevant documents or all the irrelevant ones, but to identify the most relevant documents in the top few results returned. In the context of drug discovery, testing potential drugs can be expensive and time consuming. Medicinal chemists can only test a small proportion of the compounds in their data set, so the goal is to test compounds only when the certainty of activity is high. Therefore, models with low PPV (i.e., a high false positive rate) may be less than ideal.

**Fig. 9:**
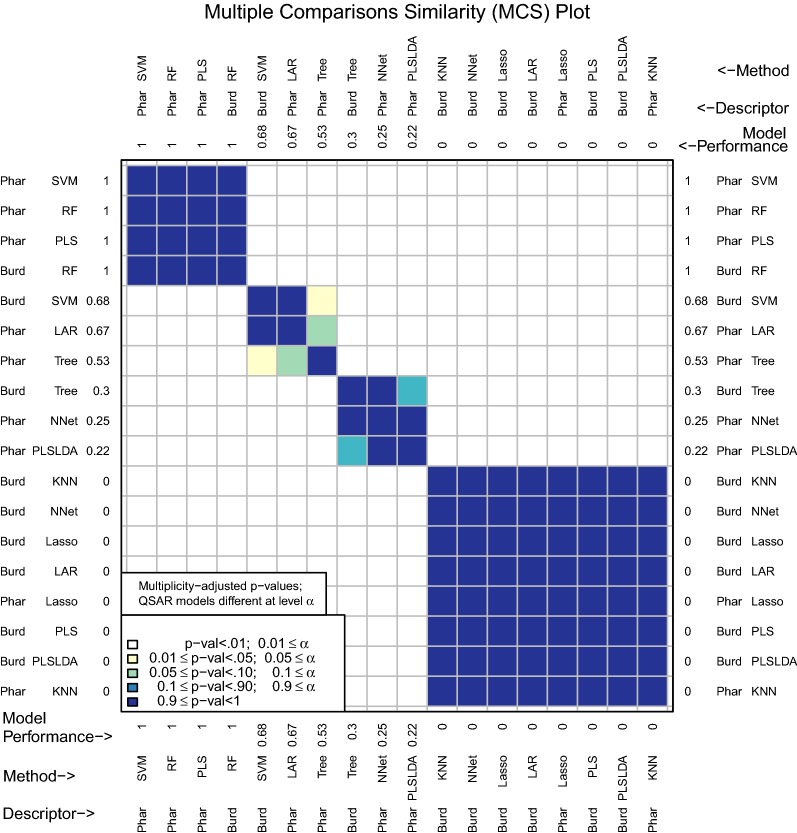
MCS plot using PPV (precision) for all compounds as the performance measure

Figure 9 shows that the best performing models according to PPV (Pharmacophore-SVM, Pharmacophore-RF, Pharmacophore-PLS, Burden Numbers-RF) all have a perfect PPV value—every compound predicted to be active was indeed active.

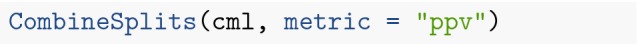



**Fig. 10:**
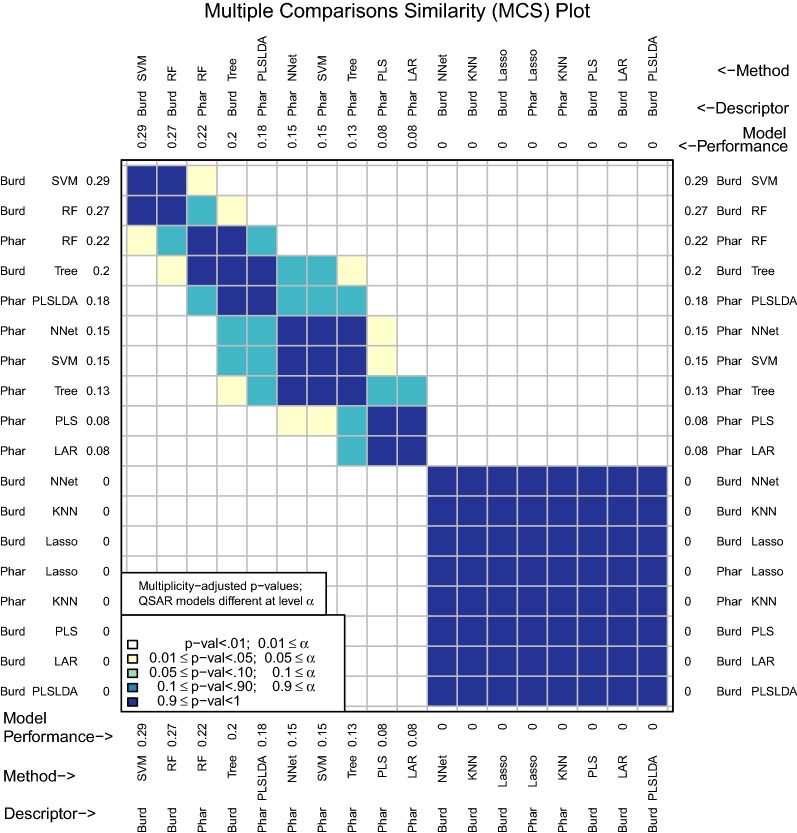
MCS plot using F1 measure for all compounds as the performance measure

When a model has both high sensitivity and PPV, this means that many of the actives in the data set were identified with a low number of false positives. The F1 measure strives to strike this balance between sensitivity and PPV. It is the harmonic mean of sensitivity and PPV. Burden Numbers-SVM and Burden Numbers-RF are the models that find the best balance, with Pharmacophore-RF being only marginally significantly different (Fig. 10).

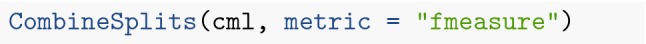



The area under the receiver operating characteristic curve (AUC) has also been implemented in chemmodlab (Fig. 1).

Table 1 summarizes the performance of the top performing D–M combinations over all performance measures considered. Burden Numbers-RF is consistently the top performer, followed by Burden Numbers-SVM. However, if a significance level of .01 were used, Pharmacophore-RF becomes a strong competitor. The fact that Pharmacophore-RF is among the best models for PPV, but is marginally significantly different for AUC, suggests that there may be a threshold for the predicted probabilities other than .5 that will lead to better prediction performance for Burden Numbers-RF and Burden Numbers-SVM.

**Table 1:** The best performing D–M combinations according to all performance measures considered

Descriptor	Model	IE 300	IE 100	Spec	Sens	Error Rate	PPV	F1	AUC
Burd	RF	$\checkmark$	$\checkmark$	$\checkmark$	$\checkmark$	$\checkmark$	$\checkmark$	$\checkmark$	$\checkmark$
Burd	SVM	$\checkmark$	$\checkmark$	$\checkmark$	$\checkmark$	$\checkmark$		$\checkmark$	
Phar	RF	$\checkmark$ –	$\checkmark$	$\checkmark$		$\checkmark$	$\checkmark$	$\checkmark$ –	$\checkmark$ –
Burd	KNN	$\checkmark$	$\checkmark$	$\checkmark$					$\checkmark$
Phar	SVM			$\checkmark$		$\checkmark$	$\checkmark$		

Check indicates the D–M combination was among the best performers according to a performance measure using a significance level of 0.05. Check minus indicates marginal significant difference between the D–M combination and the best performer (significance level between .01 and .05). Performance measures considered were: initial enhancement at 300 tests, initial enhancement at 100 tests, specificity, sensitivity, error rate, positive predictive value, F1 measure, and area under the receiver operating characteristic curve

Several performance measures have been included for continuous responses. Though root mean squared error (RMSE) is used broadly in statistics, it may not be suitable for continuous chemical assay responses used in cheminformatics. This is because under-predicting and over-predicting biological activity is equally penalized. An appropriate alternative may be initial enhancement. Other options are the coefficient of determination ($R^2$) and Spearman’s $\rho$.

### Model assessment: applicability domain

chemmodlab can also assess applicability domain using a Hotelling $T^2$ control chart. The function *ApplicabilityDomain* adapts functions in the MSQC package [41] to compute control charts for chemmodlab model objects. The control charts can identify outliers in an external data set for which predictions are desired, hence identifying compounds whose model predictions may be considered extrapolations. The definition of outlier status is made relative to the descriptor set used in *ModelTrain*. The input is a descriptor matrix contained in a chemmodlab model object and a matrix of the same descriptors computed for an external data set.

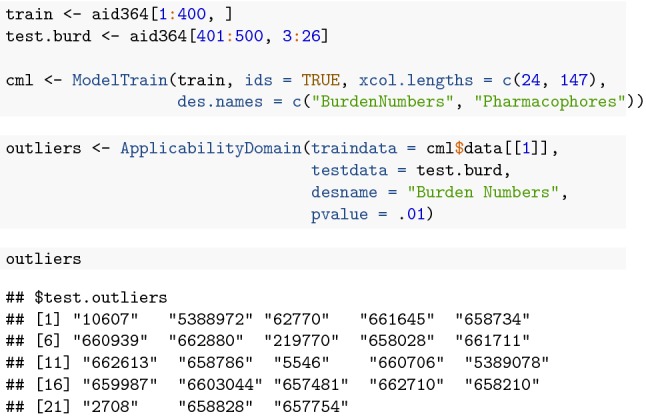



**Fig. 11:**
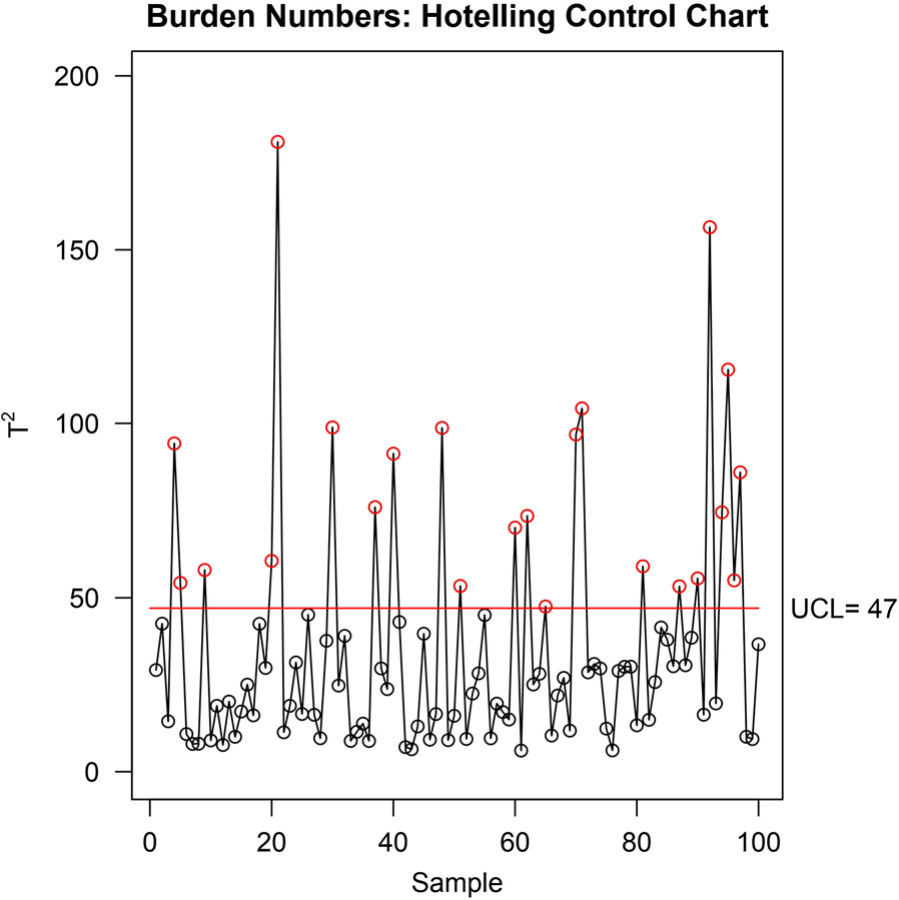
Hotelling $T^2$ control chart for an external set of aid364 compounds. Three compounds are outside of the applicability domain, based on descriptor set Burden Numbers

Hotelling $T^2$ control charts can be used to visually identify outliers (Fig. 11). For each observation in the external data set, Hotelling $T^2$ (the square of the Mahalanobis distance) is computed. This is a measure of how distant an observation’s descriptor profile is from the multivariate mean of the descriptor matrix, after adjusting for the descriptor correlation structure. This method is similar to the Euclidean distance-based approach used by Tropsha and Golbraikh [42], but there is one important advantage. Observations that may not appear to be outliers according to Euclidean distance from the mean can still be identified as outliers by Hotelling $T^2$ if they violate the correlation structure of the descriptor matrix (see [43]). Several cheminformatics studies have illustrated the utility of this approach [43, 44]. A significance level can be provided to compute an upper control limit (UCL) for the Hotelling $T^2$ statistic. Details on how these thresholds are computed for the external set can be found in [45]. Observations beyond the control limit are considered outliers, and are outside the applicability domain. The user may wish to omit these from prediction.

A limitation of this approach is that a $p \times p$ covariance matrix needs to be estimated, where *p* is the number of descriptors. This is challenging when the descriptor space is high dimensional. However, other distance-based methods also suffer in high dimensional spaces due to the curse of dimensionality.

## Conclusions and future directions

chemmodlab provides a comprehensive collection of methods for fitting and assessing machine learning models. While these methods have been selected for their utility to the cheminformatics community, they can be applied to any data set with binary or continuous variables. These methods are applicable to a wide range of research areas, and some model assessment approaches (the MCS plot, continuous valued accumulation curves) are novel additions to the collection of assessment approaches for machine learning methods available in R. The functions in chemmodlab aim to enable researchers to try many different model fitting and assessment procedures with ease.

chemmodlab has many future directions in store. Parallel processing will be utilized so that model fitting for different descriptor sets, splits, and cross validation folds can be done in parallel. We also plan to interface chemmodlab with caret, employing their functions for model tuning during our model fitting procedure. Support for categorical variables with more than two levels will be arriving soon. We will also incorporate more extensions to the accumulation curve as this approach is used extensively in drug discovery. Extensions will include the area under the accumulation curve as an assessment measure and the construction of a mean accumulation curve over multiple splits. Error bars can be plotted for these curves so that significant differences between D–M combinations can be analyzed across the entire curves. Additional graphical output is planned, e.g., to provide receiver operating characteristic curves and precision recall curves along with accumulation curves, per user request. Finally, more modeling methods will be added as we identify those with appeal to our user base. Support for a particular model may be requested here: https://github.com/jrash/chemmodlab/issues.

## Abbreviations

ANOVA: analysis of variance
AUC: area under the receiver operating characteristic curve
D–M: descriptor set and modeling routine
ENet: elastic net
KNN: k-nearest neighbors
LAR: least angle regression
Lasso: lasso
LOOCV: leave-one-out-cross-validation
MCS: multiple comparisons similarity
NNet: neural networks
PCR: principal components regression
PLS: partial least squares
PLSLDA: partial least squares linear discriminant analysis
PPV: positive predictive value
QSAR: quantitative structure–activity relationship
$R^2$: coefficient of determination
RF: random forest
Ridge: ridge regression
RMSE: root mean squared error
RPart: recursive partitioning
SVM: support vector machines
Tree: recursive partitioning
